# Depression and Anxiety on Twitter During the COVID-19 Stay-At-Home Period in 7 Major U.S. Cities

**DOI:** 10.1016/j.focus.2022.100062

**Published:** 2022-12-22

**Authors:** Danielle Levanti, Rebecca N. Monastero, Mohammadzaman Zamani, Johannes C. Eichstaedt, Salvatore Giorgi, H. Andrew Schwartz, Jaymie R. Meliker

**Affiliations:** 1 Undergraduate Studies, Cornell University, Ithaca, New York; 2 Renaissance School of Medicine, Stony Brook University, Stony Brook, New York; 3 Department of Computer Science, College of Engineering and Applied Sciences, Stony Brook University, Stony Brook, New York; 4 Department of Psychology, School of Humanities and Sciences, Stanford University, Palo Alto, California; 5 Department of Computer and Information Science, University of Pennsylvania, Philadelphia, Pennsylvania; 6 Program in Public Health, Department of Family, Population, & Preventive Medicine, Renaissance School of Medicine, Stony Brook University, Stony Brook, New York

**Keywords:** Social media, mental health, stay-at-home order, coronavirus

## Abstract

**Introduction:**

Although surveys are a well-established instrument to capture the population prevalence of mental health at a moment in time, public Twitter is a continuously available data source that can provide a broader window into population mental health. We characterized the relationship between COVID-19 case counts, stay-at-home orders because of COVID-19, and anxiety and depression in 7 major U.S. cities utilizing Twitter data.

**Methods:**

We collected 18 million Tweets from January to September 2019 (baseline) and 2020 from 7 U.S. cities with large populations and varied COVID-19 response protocols: Atlanta, Chicago, Houston, Los Angeles, Miami, New York, and Phoenix. We applied machine learning‒based language prediction models for depression and anxiety validated in previous work with Twitter data. As an alternative public big data source, we explored Google Trends data using search query frequencies. A qualitative evaluation of trends is presented.

**Results:**

Twitter depression and anxiety scores were consistently elevated above their 2019 baselines across all the 7 locations. Twitter depression scores increased during the early phase of the pandemic, with a peak in early summer and a subsequent decline in late summer. The pattern of depression trends was aligned with national COVID-19 case trends rather than with trends in individual states. Anxiety was consistently and steadily elevated throughout the pandemic. Google search trends data showed noisy and inconsistent results.

**Conclusions:**

Our study shows the feasibility of using Twitter to capture trends of depression and anxiety during the COVID-19 public health crisis and suggests that social media data can supplement survey data to monitor long-term mental health trends.

## HIGHLIGHTS

• Twitter depression and anxiety scores were elevated across 7 U.S. cities.
• Twitter depression trends were aligned with national COVID-19 case trends.
• Twitter anxiety trends were consistently elevated throughout the pandemic.
• Google search trends data showed noisy and inconsistent results.
• Twitter can supplement surveys to assess psychological well-being in populations.

## INTRODUCTION

After the advent of coronavirus disease (COVID-19) in the U.S. in early 2020, the U.S. implemented multiple measures in an attempt to slow down the spread of this highly transmissible virus, including social distancing, mask wearing, and closure of nonessential businesses and schools. Between March 1, 2020 and May 31, 2020, 42 states and territories issued stay-at-home orders.^1^ Although the duration and enforcement of these orders varied, they shared common quarantine-like mandates of limiting population movement for essential tasks to lower COVID-19 transmission, resulting in an unprecedented and widespread shift in the daily lives of most U.S. residents to be highly isolated.^2^

Social isolation during the stay-at-home period presented a particular threat to well-being. A wide body of work has established that social relationships are among the leading determinants of subjective well-being.^3^ We are a fundamentally social species; the physical presence of others regulates our emotions, including those occurring in response to threat.^4^ By contrast, loneliness, the result of insufficient quality and quantity of social relationships, has detrimental impacts on mental health^5^ as well as physical health and lifespan in general.^6^ For example, among older adults, loneliness and isolation may be predictors for symptoms of depression and anxiety.^7^^,^^8^

In addition to social isolation, the experience of the pandemic has been marked by emotions associated with sympathetic arousals, such as fear, worry, and agitation, with their behavioral manifestations such as rumination and hypervigilance.^9^ Sources of fear include contracting the virus, losing loved ones, lack of resources, and economic struggles as well as uncertainty about the future. Both negative emotions and social isolation during the pandemic may contribute to poor mental health outcomes.

Previous work has identified negative mental health outcomes in earlier epidemics requiring quarantine, including severe acute respiratory syndrome, Middle East respiratory syndrome, and Ebola.^10^ In a study of healthcare workers with potential severe acute respiratory syndrome exposure, quarantined individuals showed increased symptoms of acute stress disorder, anxiety under specified conditions, irritability, insomnia, poor concentration, and decreased quality of their work compared with those who were not quarantined.^11^ Another study examined post-traumatic stress symptoms in adults and children in quarantine and found that those in quarantine faced significantly higher post-traumatic stress scores than those who had not been quarantined.^12^

Several scholars predicted negative mental health outcomes during the COVID-19 pandemic and stay-at-home periods because of loneliness and isolation and concluded that increased mental health support would be particularly necessary to prevent increased suicides, depression, and anxiety.^13^^,^^14^ Indeed, recent studies have noted the increased prevalence of mental health struggles in the U.S. during COVID-19 through survey data. For example, 1 survey of U.S. adults in late June 2020 showed that 40% of respondents were struggling with mental health or substance abuse issues during the pandemic; 13% of respondents reported anxiety or depression symptoms.^15^ An international study during the pandemic that collected survey data from 78 countries showed that poor mental health was prevalent among 10% of survey respondents and that moderate mental health was prevalent among 50%.^16^ A recent review of 19 mental health‒related studies during the pandemic found a prevalence of depressive symptoms of 15%‒48% compared with a prepandemic prevalence of 4%‒7%.^17^ In the same review, the prevalence of anxiety during the pandemic was identified as ranging between 6% and 51%.

There are many strengths to these surveys; however, they are limited in (1) time (moments or weeks measured), (2) sample (number of residents represented), and (3) scope (narrowly limited to anxiety and depression outside of the rapidly changing everyday concerns during COVID-19 and stay-at-home onset). Publicly available Twitter data have been shown to provide an alternative window into population mental health insights and how those trends change over time.^18^^,^^19^ Although imperfect samples, previous work has shown high convergence between Twitter-based psychological assessments and representative mental and physical health outcomes.^20^, ^21^, ^22^ In addition, they provide a more readily available source of data should we need to identify social and psychological trends in a future pandemic.

In this study, we seek to characterize the relationship between stay-at-home orders because of COVID-19 and anxiety and depression in 7 major U.S. cities utilizing Twitter data. We selected 7 U.S. cities located around the country to examine the role of local case trends and stay-at-home orders. We compare monthly data on anxiety and depression from January to September 2020 with data from the same period in 2019.

## METHODS

### COVID-19 Data

COVID-19 case counts in the states of Arizona, California, Florida, Georgia, Illinois, New York, and Texas were captured from the *New York Times* COVID-19 website: https://www.nytimes.com/interactive/2021/us/covid-cases.html. Dates of statewide stay-at-home orders in 2020 were recorded from sources listed in Appendix Table (available online) and cross-referenced with articles in local newspapers in each state and state-issued executive orders.

### Twitter Data

We compiled monthly Twitter-based data between January and September of 2019 and 2020 in 7 U.S. cities: Atlanta, GA; Chicago, IL; Houston, TX; Los Angeles, CA; Miami, FL; New York City, NY; and Phoenix, AZ. These cities were selected for analysis owing to their large populations and varied COVID-19 response protocols and stay-at-home orders. To build the Twitter data set, we began with a random sample of 1% of all publicly available tweets from January to March 2020. The tweets in this random sample were mapped to U.S. cities on the basis of free-response location fields and geographic coordinates. This approach was found to agree with human judgments of the intended city 94% of the time.^20^ Next, we took all Twitter users mapped to the 7 cities mentioned earlier and collected each user's tweet history dating back to January 1, 2019. We then restricted the tweet sample to between January 1 and September 30 of both 2019 and 2020. Following thresholds set by the County Tweet Lexical Bank,^23^ a large open-source database of U.S. location‒mapped Twitter data, each user posted at least 30 tweets, and we randomly sampled 10,000 users per city. This resulted in a total of 56,411,200 tweets from 70,000 Twitter users. Full statistics are reported in Appendix Table 2 (available online), with example tweets in Appendix Table 3 (available online).

**Artificial intelligence‒based language assessment.** The text of the tweet was input into 2 artificial intelligence (AI)-based assessments of depression and anxiety developed by Schwartz et al.^24^^.^^25^ These models required 3 types of linguistic features: (1) relative frequencies of words and phrases, (2) binary indicators of words and phrases, and (3) topic prevalence scores. Words and phrases are sequences of 1 to 3 words in a row. Their relative frequency was recorded by counting each word or phrase mentioned and dividing by the total number of words or phrases mentioned by the Twitter user. The binary indicator for words and phrases simply indicated whether each word or phrase is present (1) or not (0).

**Analytic strategy.** To apply the approach of Schwartz et al.,^24^ all word, phrase, and topic features were extracted for each Twitter user for each month in the data set. Once extracted, all features were averaged across all Twitter users within each of the 7 cities, resulting in city-level word, phrase, and topic scores for each month. We then applied the depression and anxiety AI assessments to each of the city‒month feature sets and then calculated the difference between the 2019 and 2020 scores. We then standardized the monthly scores for both depression and anxiety for each of the 7 cities by subtracting the mean and dividing by the SD across all city‒month data points across 2019 (baseline) and 2020 (also known as a *z*-score). All analyses, described earlier, were performed using the open-source python package DLATK (Differential Language Analysis ToolKit).^25^

### Google Trends Data

As a secondary analysis, we also compiled Google Trends deidentified data at the city level. This is a more simplistic method than the AI-based assessment we used for Twitter. This method relies on weekly internet search term volumes, which represent the entire number of searches standardized on a 0–100 scale: 0 indicates no searches in a given time frame for a given term, and 100 represents the maximum number of searches in a given time frame for a given term. Search data were collected for the same 7 U.S. cities. Location at the time of search was determined by Google through user location data, including Internet Protocol address and GPS data, among others.

Search terms were generated on the basis of terms used in previous work evaluating mental health through Internet search trends and were tailored to specifically identify patients with mental health issues.^26^^,^^27^ *Keywords with insufficient data*, defined as a search volume of 0 for any month for 2 or more cities for a given term, were excluded from our analysis. Search terms included the following mental health‒related terms or phrases: *Anxiety, Signs of anxiety, Symptoms of anxiety, Depression, Signs of depression, Symptoms of depression*, and *Panic attack*. Trends for anxiety and depression are presented in the Appendix Figures (available online); the other search terms yielded similarly ambiguous results and are not shown. We present relative search volume graphs at the monthly level per city. We calculated the monthly difference in relative search volume scores for each of the 7 cities, comparing the average from 2020 with the city‒month average from 2018 and 2019. Owing to minor changes in data when accessed on different days with the same specifications, for consistency, all data were gathered on February 1, 2021.^28^

This work was approved as exempt, nonhuman subjects by an academic IRB. This approval was granted because it utilized publicly available and aggregate data from Google Trends and Twitter.

## RESULTS

**Figure 1:**
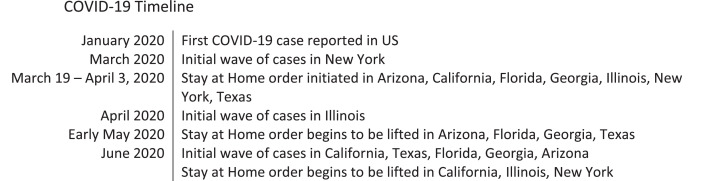
Timeline of major COVID-19‒related events in 2020, as relevant to the 7 cities under study.

A timeline of U.S. COVID-19 waves of cases in the 7 states and statewide stay-at-home orders is shown in Figure 1. In Appendix Figure 1 (available online), we see the locations of the 7 cities on a U.S. map, and in Appendix Figure 2 (available online), we see that cases began to sharply increase in New York in March and in Illinois in April 2020 but did not sharply increase until June and July 2020 in the other locations: Georgia, Texas, California, Arizona, and Florida. Stay-at-home orders were adopted in the 7 states in the spring in response to the national spread of COVID-19 but were removed by early May in Arizona, Texas, Florida, and Georgia before the increased number of cases in some of the states in June.

### Depression

**Figure 2:**
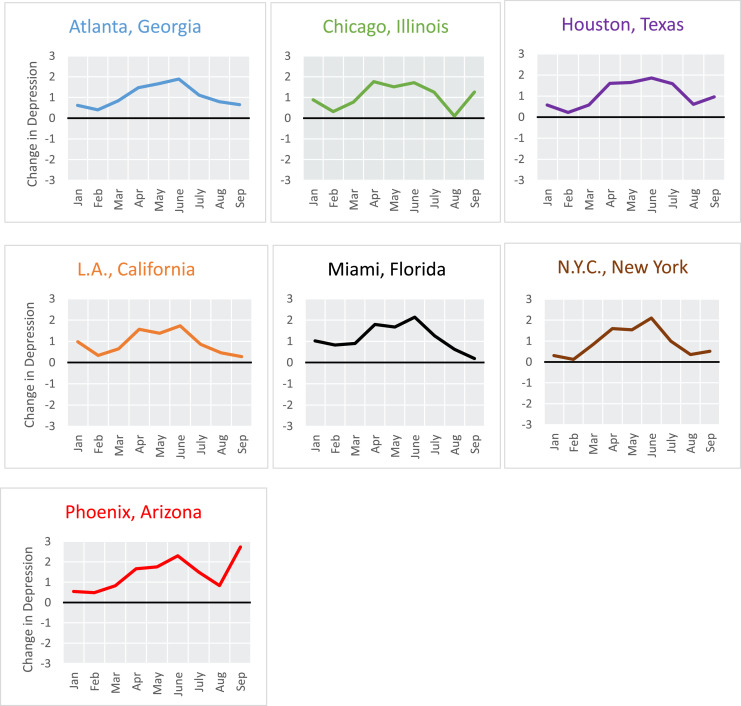
Twitter trends for change in depression, the difference between 2020 and 2019, by city and month, January–September *Note:* Units of Twitter scores reflect an SD change between 2020 and 2019. A change equal to 1 reflects a 1 SD change in depression score. Apr, April; Aug, August; Feb, February; Jan, January; L.A., Los Angeles; Mar, March; N.Y.C., New York City; Sep, September.

Twitter-based depression scores resulted in trends of increased depression during the early phase of the pandemic, with a peak in early summer and a subsequent decline in late summer in each of the 7 cities, although Phoenix showed increasing depression rates in September 2020 (Figure 2). Depression trends appeared to be more in sync with the national prevalence of COVID-19 case trends (Appendix Figure 2, available online) than with local trends of cases or stay-at-home orders in individual states.

### Anxiety

**Figure 3:**
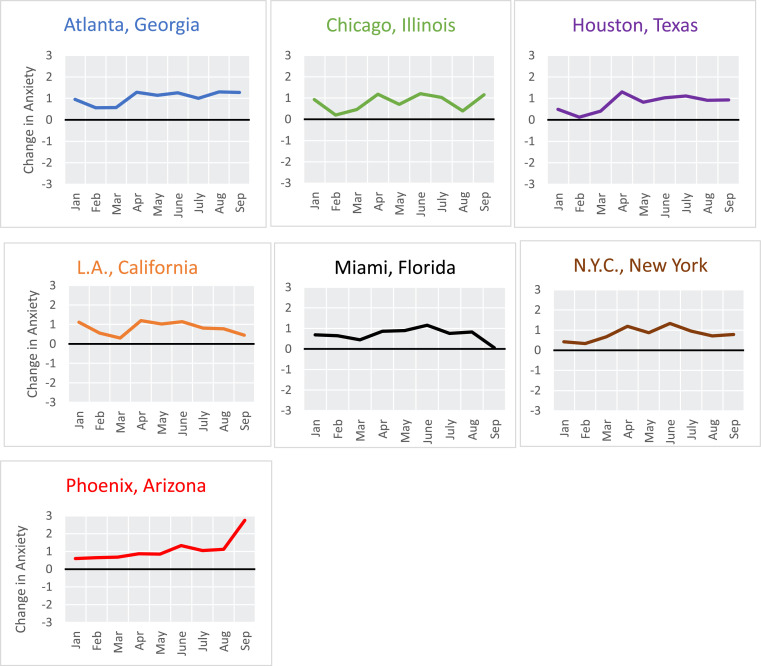
Twitter trends for change in anxiety, the difference between 2020 and 2019, by city and month, January–September *Note:* Units of Twitter scores reflect an SD change between 2020 and 2019. A change equal to 1 reflects a 1 SD change in anxiety score. Apr, April; Aug, August; Feb, February; Jan, January; L.A., Los Angeles; Mar, March; N.Y.C., New York City; Sep, September.

Anxiety levels based on Twitter data were overall elevated when compared with those in the previous year, most consistently elevated beginning in April and continuing into the Autumn, with Phoenix again showing an increase over all previous values in September (Figure 3). Most cities saw an initial peak in anxiety in April, coincident with national COVID-19 case trends, and remained elevated throughout the summer months, regardless of local case trends or stay-at-home orders.

### Secondary Analysis: Google Search Terms

In the secondary analysis using relative search volume of Google search terms, no strong patterns were observed for depression- or anxiety-related searchers across cities or over time (Appendix Figures 3 and 4, available online). All cities and search terms that showed considerable increases also showed considerable decreases at other times of the year, although with inconsistent patterns.

## DISCUSSION

This study employed a qualitative comparison of mental health trends over the pandemic on the basis of Twitter posts and Google search queries. Our study found overall increases in depression and anxiety expressed on Twitter throughout the early pandemic (until September 2020). The Twitter analysis was consistent with our hypothesis of increased depression and anxiety in the early stages of the pandemic, which is also consistent with survey data during the same period.^15^, ^16^, ^17^ According to Twitter data, depression was higher earlier and resolved toward the end of the summer, whereas anxiety increased in April and stayed elevated into the autumn. We had hypothesized that these trends would vary by city on the basis of local COVID-19 case trends or stay-at-home orders, but this was not the case; the trends were largely consistent across the cities, reflecting the national milieu and not local factors.

Our secondary analysis relied on a simpler approach to evaluating patterns of search terms in Google Trends data. These data did not show trends similar to those of the Twitter data; when compared with the 2019 baseline period, the difference from 2020 scores appeared noisy with frequent peaks and troughs, and not showing a clear association with COVID-19 trends. The figures appear to approximate fluctuations from normal variation. Recent studies used Google Trends data to characterize mental health‒related outcomes during the pandemic similar to what we have done in this study. Results have been mixed, with some showing Google Trends data matching the anticipated increases in anxiety or depression^29^ and others not reporting increases in depression or anxiety.^30^, ^31^, ^32^ We also note a recent report indicating that Google Trends data do not appear to be a useful indicator of changing the levels of population mental health during a public health emergency.^33^ Previous work also found that some approaches to using Google Trends for forecasting suicide rates were not very accurate.^28^ By contrast, Twitter data seem to aid in predicting infectious diseases such as influenza^34^ and more reliably capture mental health trends and be more aligned with survey data during the early phase of the pandemic.^35^^,^^36^ We think this is because many on Twitter share their daily thoughts, feelings, and behaviors,^19^ whereas Google Trends capture what populations are searching for.^37^ Conceivably, Google Trends data may be suitable for other use cases, such as viral prevalence surveillance as people search for the particular symptoms they are encountering. For example, some previous work has shown that people mention more influenza symptoms in areas at times with higher rates of influenza.^34^

We did not observe strong influences of state trends in case rates or stay-at-home orders. By and large, the trends picked up by Twitter appear to follow the nationwide COVID-19 case rates, for example, with increases in depression from April through July in most cities even before they experienced local increases in cases and after local stay-at-home orders ended. Mortality tended to follow cases on a 2-week delay in 2020 (before vaccinations and better treatments), with mortality peaks generally reflecting the case peaks (Appendix Figure 2, available online); therefore, local mortality trends also were not correlated with local changes in depression or anxiety. This may point to the fact that national (rather than local) reporting of pandemic developments may offer the most salient psychological context that the mental health rates reflect.

### Limitations

A limitation of the study is the observational nature of our work as well as the inability to distinguish between the impact of policy and the impact of the pandemic. We do not have the ability to control for individual-level confounders, and we lack knowledge of the demographics of participants. Race, ethnicity, SES, age, and a multitude of other factors may contribute to individuals’ responses to the pandemic. We also acknowledge that the protests and social unrest in June 2020 could influence feelings of depression and/or anxiety, and that is not considered in this study. We also cannot distinguish between mental health issues because of pandemic stressors (e.g., fear of illness, fear of losing loved ones, and so on) and mental health issues resulting from isolation and social distancing policies. It is likely that both policy and pandemic have contributed to overall mental health, and our work is not equipped to distinguish the two, if separable.

Although Twitter is an important source of big data, it also has limitations in utility. One possible problem with Twitter, Google Trends, or other social media platforms is the issue of ambiguities in language use. One limitation is that words can carry multiple meanings such that an individual writing *great* might not be referring to how they feel but rather using it as an adjective as in a great sadness. The AI-based approach we use partially solves this by not assuming relationships between words and depressivity but rather learning it from examples.^24^^,^^38^ Furthermore, social media are known to skew young and thus introduce a selection bias. On average, most Twitter users in this sample have posted 700–900 tweets (Appendix Table 2, available online), which is roughly 350–450 tweets per year. In general, social media‒based language assessments rely on users posting a minimum number of words (e.g., 500‒1,000 words) to derive stable estimates. Thus, these findings may not generalize to populations who are not as active on Twitter. Finally, there may be errors in the geolocation process. For example, we assign a location to each Twitter user's timeline on the basis of a single tweet. In addition, we assign a static location and do not consider that tweets may originate from different locations (e.g., traveling) or the fact that these users may have moved. There may also be a selection bias because Twitter users who do not turn on location data or self-report their location in their profile will not be in the sample. Taken together, these issues (errors in the geolocation process, errors in the mental health estimates, selection biases, and high minimum words and tweet thresholds) should only make the process of estimating community attributes more difficult. Nevertheless, past work has shown on average that community lexical variance does match accepted community measurements: people do tweet more influenza symptoms in areas at times with higher rates of influenza,^34^ positive emotion words are mentioned more in areas surveying higher life satisfaction,^20^^,^^22^ and individuals with medical diagnoses of depression are more likely to talk about depressive symptoms (e.g., low mood, somatic pain, hostility, and loneliness) on social media.^3^

## CONCLUSIONS

Our study observes Twitter trends over a prolonged timeframe during the COVID-19 pandemic to shed insights on possible mental health outcomes of a public health crisis. These data are not meant to replace survey data but can supplement questionnaires to help identify the long-term psychological impacts of social distancing during pandemics. Policy makers and other officials should plan for such events and consider both physical and psychological aspects of human health when deciding on effective public planning. With increased information about the pandemic's effect on mental health, either owing to isolation or owing to other concerns surrounding the pandemic, we will be better equipped to provide resources and mitigate these public health concerns in the future.

## Appendix materials

Image, application 1

## Footnotes

Supplementary material associated with this article can be found in the online version at doi:10.1016/j.focus.2022.100062.
