# Supplementary material for: Depression and Anxiety on Twitter During the COVID-19 Stay-At-Home Period in 7 Major U.S. Cities
Source: AJPM Focus. 2022 Dec 22;2(1):100062. doi: 10.1016/j.focus.2022.100062 (PMC9773738; doi:10.1016/j.focus.2022.100062)
Supplement: Supplementary file 1 [file mmc1.docx]

Appendix Figure 3. 7 Cities Under Study on a US Map


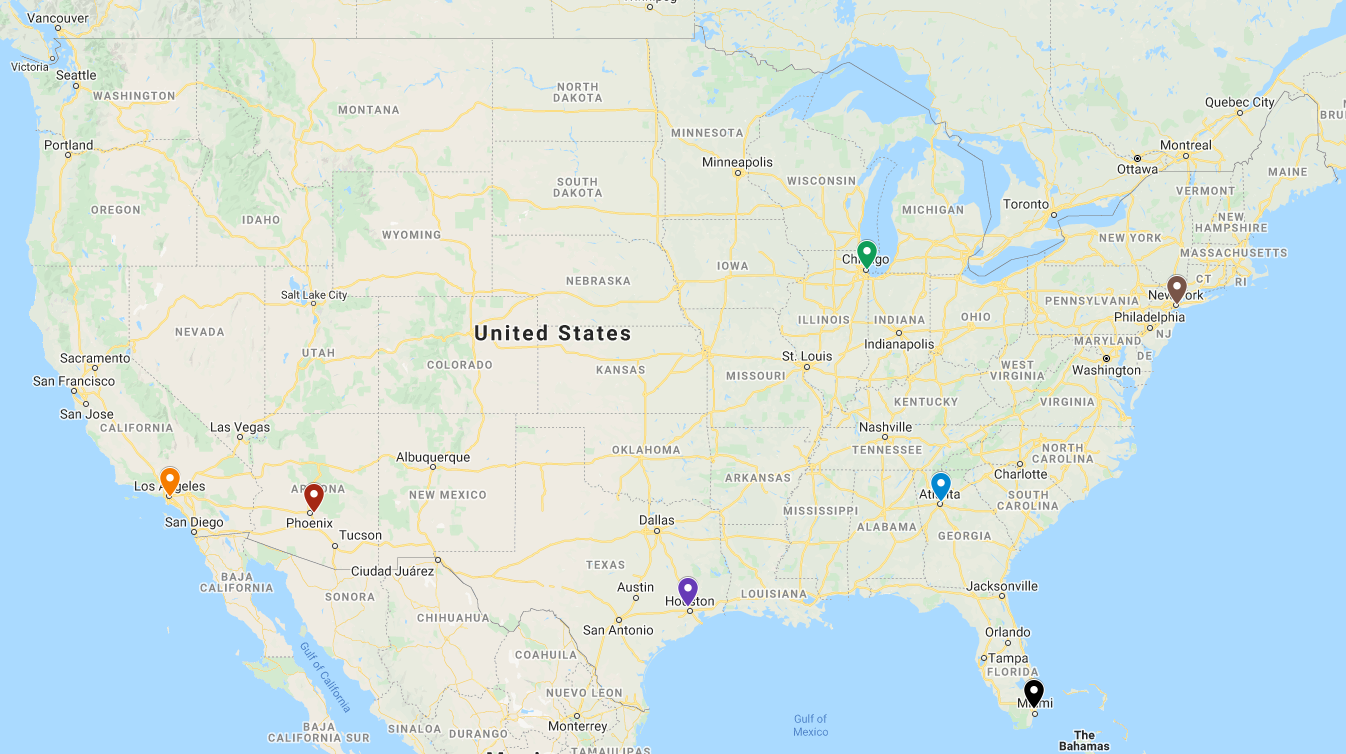


Figure generated with Google Maps

Appendix Table 1. Dates for Stay at Home Orders in each State

| State | Dates | Data Source |
| --- | --- | --- |
| Arizona | March 29-April 30 | https://www.huschblackwell.com/arizona-state-by-state-covid-19-guidance |
| California | March 19-June 12 | https://www.huschblackwell.com/california-state-by-state-covid-19-guidance |
| Florida | April 3- May 4 | https://www.huschblackwell.com/florida-state-by-state-covid-19-guidance |
| Georgia | March 23-April 27 | https://www.huschblackwell.com/georgia-state-by-state-covid-19-guidance |
| Illinois | March 19-May 29 | https://www.huschblackwell.com/illinois-state-by-state-covid-19-guidance |
| New York | March 22-May 29 | https://www.huschblackwell.com/new-york-state-by-state-covid-19-guidance |
| Texas | April 2-April 30 | https://www.huschblackwell.com/texas-state-by-state-covid-19-guidance |

Note, the reason we chose not to provide government sources is that they are often piecemeal, with one documenting the initial date, another documenting an extension, and another documenting the end of the stay at home order. It becomes quite a lot of weblinks to document this at the state level; for this reason we have linked to other sources which coalesced the information in one place. However, we have verified the information by cross referencing with the state issued material.

Appendix Table 2. Twitter Data Statistics

| **City** | **Number of Users** | **Number of Tweets** | **Mean (SD) tweets per user** |
| --- | --- | --- | --- |
| Atlanta | 10,000 | 8,974,099 | 905 (633) |
| Chicago | 10,000 | 7,804,010 | 780 (565) |
| Houston | 10,000 | 8,576,660 | 860 (631) |
| Los Angeles | 10,000 | 8,895,580 | 892 (636_ |
| Miami | 10,000 | 6,870,411 | 686 (612) |
| New York City | 10,000 | 8,529,282 | 852 (623) |
| Phoenix | 10,000 | 6,761,158 | 678 (617) |

Appendix Table 3: Example Twitter Posts High in Depression and Anxiety, Taken from a Random Subsample of 10,000 Tweets. Posts are Paraphrased from the Original Posts to Preserve Anonymity.

| *Depression* | |
| --- | --- |
|  | If I'm acting funny it's because I have financial stress, plus some home issues. My boyfriend is in the hospital and I don't know which hospital. I'm not one who copes well, so please ignore my dramatic posts. |
|  | I met a dude that grew up in Philly. I said that whenever I thought about Philly I thought about Hall and Oates. He laughed and said "I used to see Hall around all the time". He was super friendly until you brought up Oates. |
|  | There are two kinds of depression: tired of living and can something just take me out through the frontal lobe. Super quick! I'm the latter. So tired and not crying for help. |
|  | It's our stress time of year. New Years follow by my birthday followed by ANOTHER BIRTHDAY! He's stressed and I'm excited. |
|  | The police are making arrests now? What about when people were out here "protesting" that they couldn't get a haircut? Lives were lost! We are upset! Yet these "protests" were putting lives at risk by not social distancing |
| *Anxiety* | |
|  | I really regret my last relationship... uuugghhhh. I was down and out when we met, depressed and lots of negative emotions. I would've settled for anyone. So embarassing, for real. |
|  | The debates were not moderated well. It's like a reality show aimed at drama ratings. Garbage fake news trying to diminish candidates they don't like. |
|  | Normalizing how parents abuse their children is not right. My family never did this. The worst thing that happened to me was when I snuck out for the night. I love my family but that really hurt. |
|  | Let me know if you have a loved-one who can't go grocery shopping or needs help. I'll go shopping for them. It's so very disgusting how out of hand people are these days. |
|  | Racism is alive. It's so disgusting and we all must acknowledge it. But that's not enough. Hating racism is not enough. You must do something about it. Something must be done about injustice happening all the time. |

Appendix Figure 4. US case trends of COVID-19, March-December 2020 in 7 States and the US.


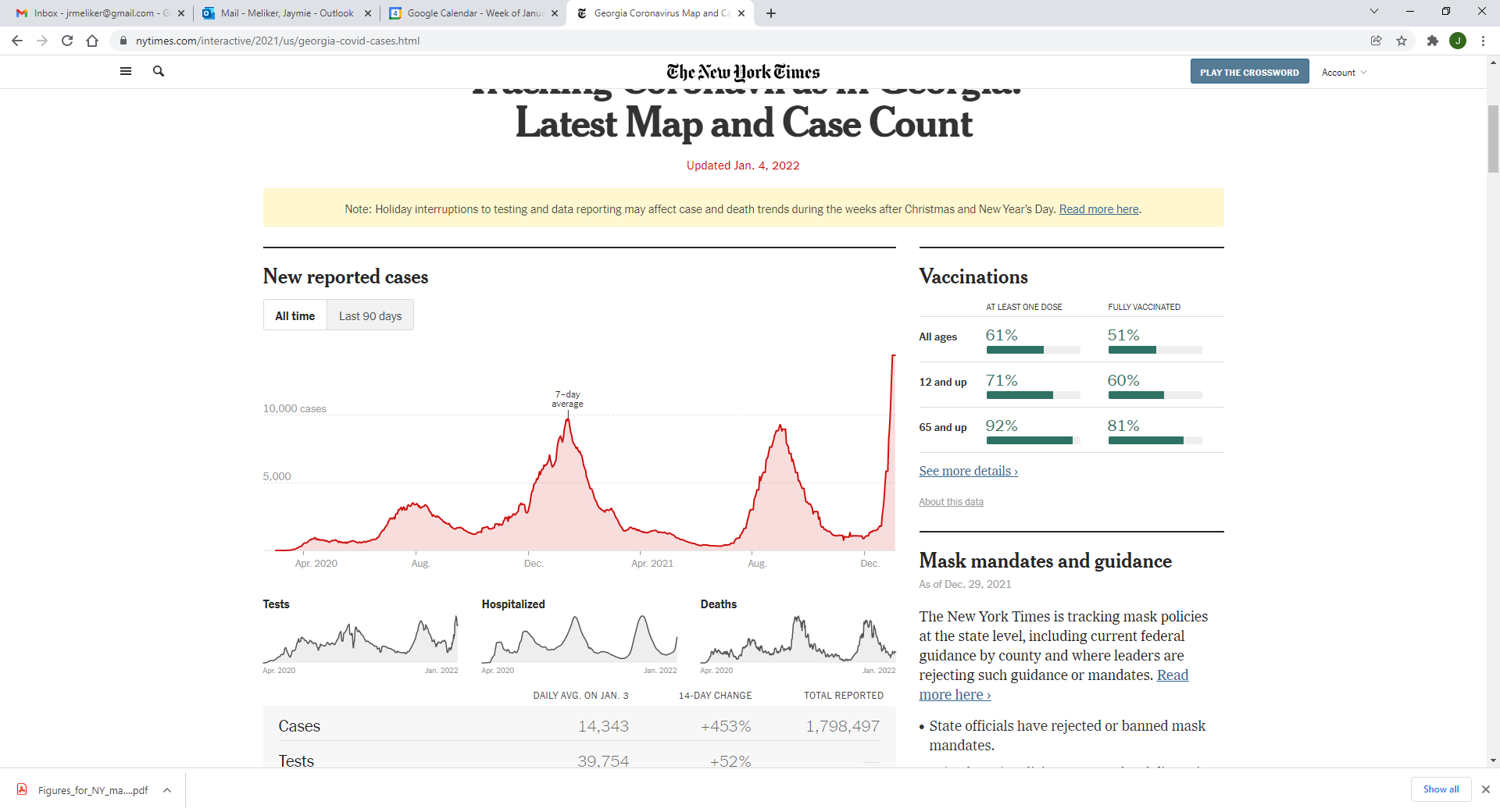

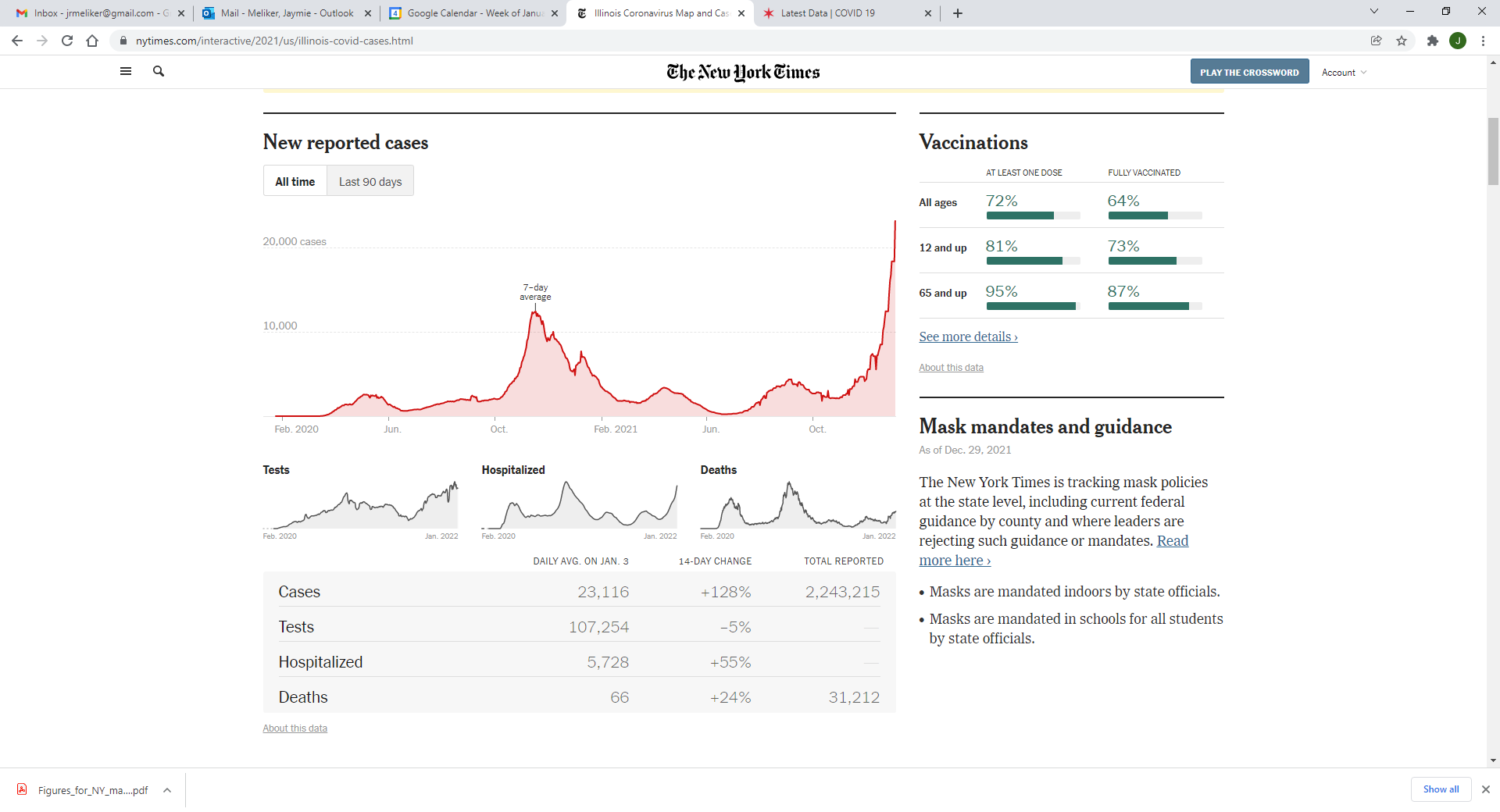

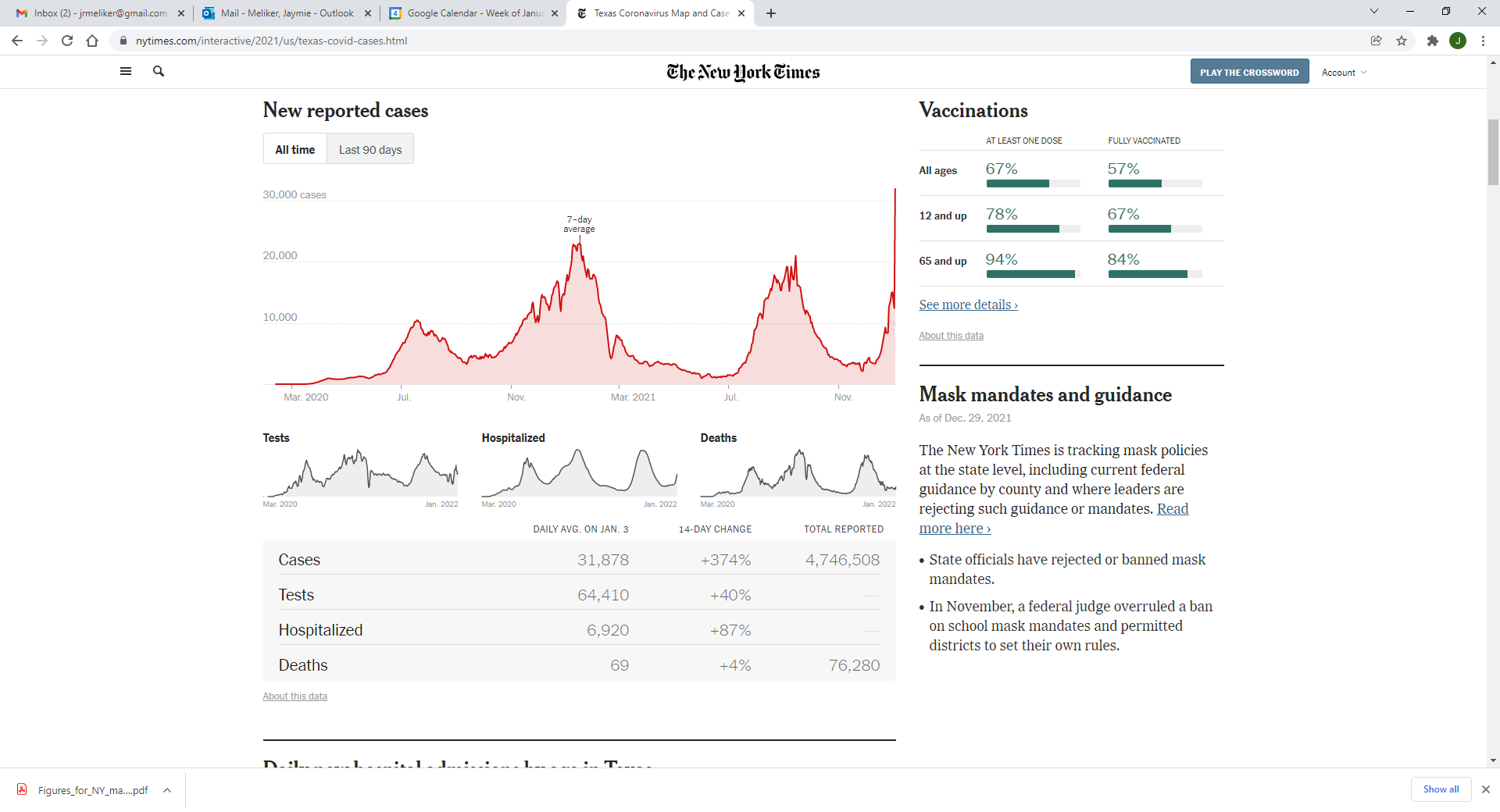


**Georgia**

**Texas**

**Illinois**


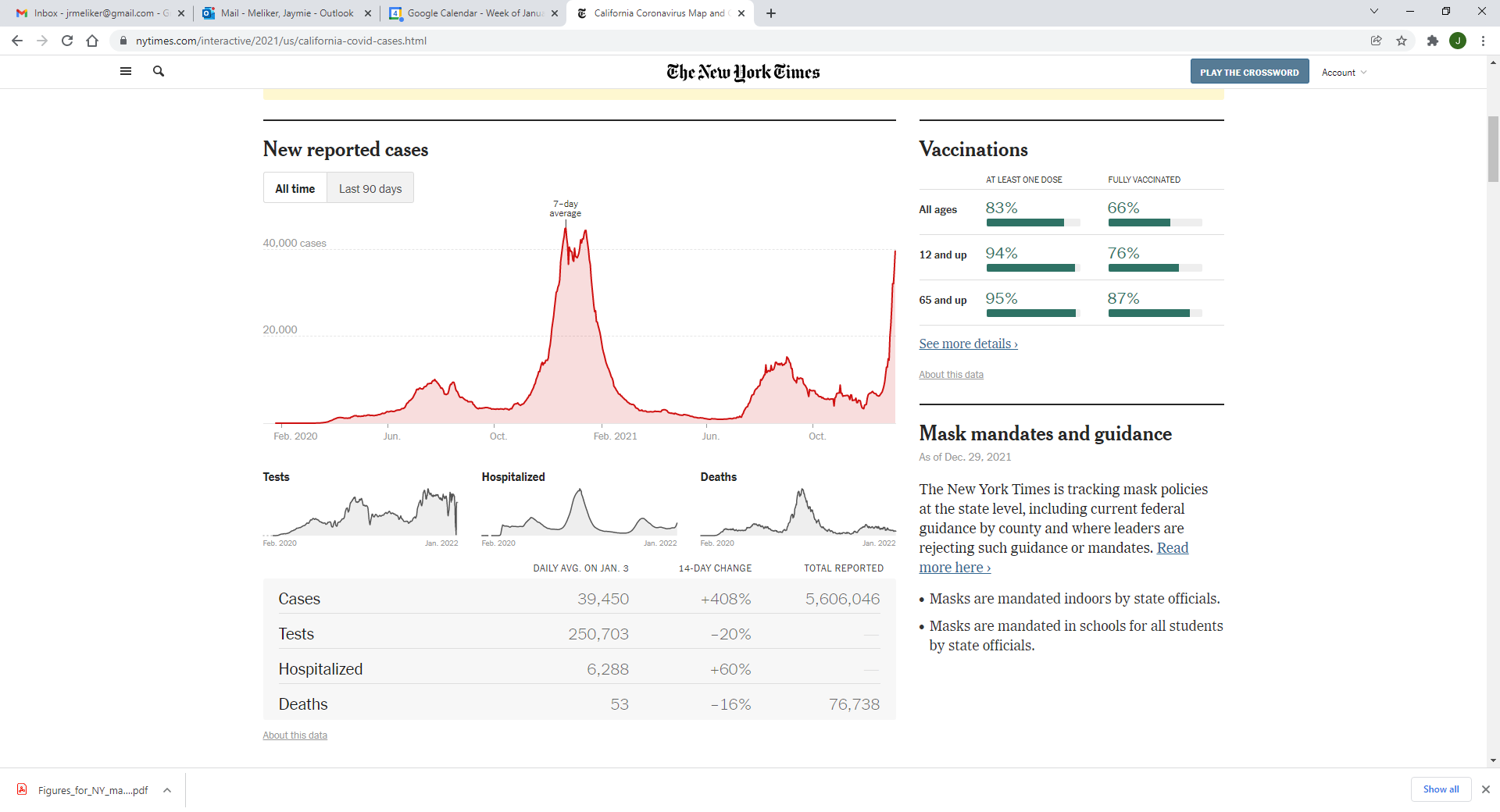

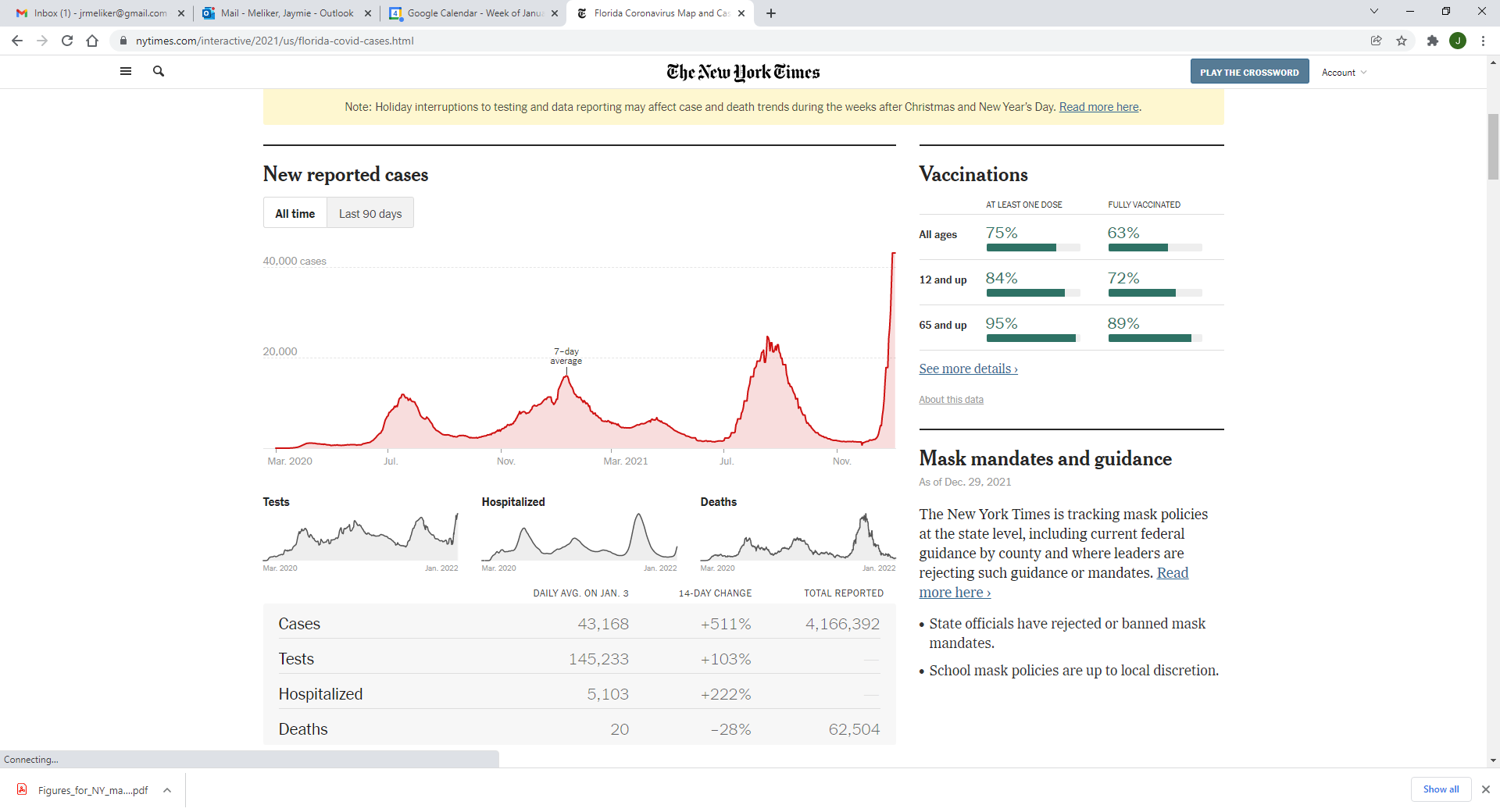

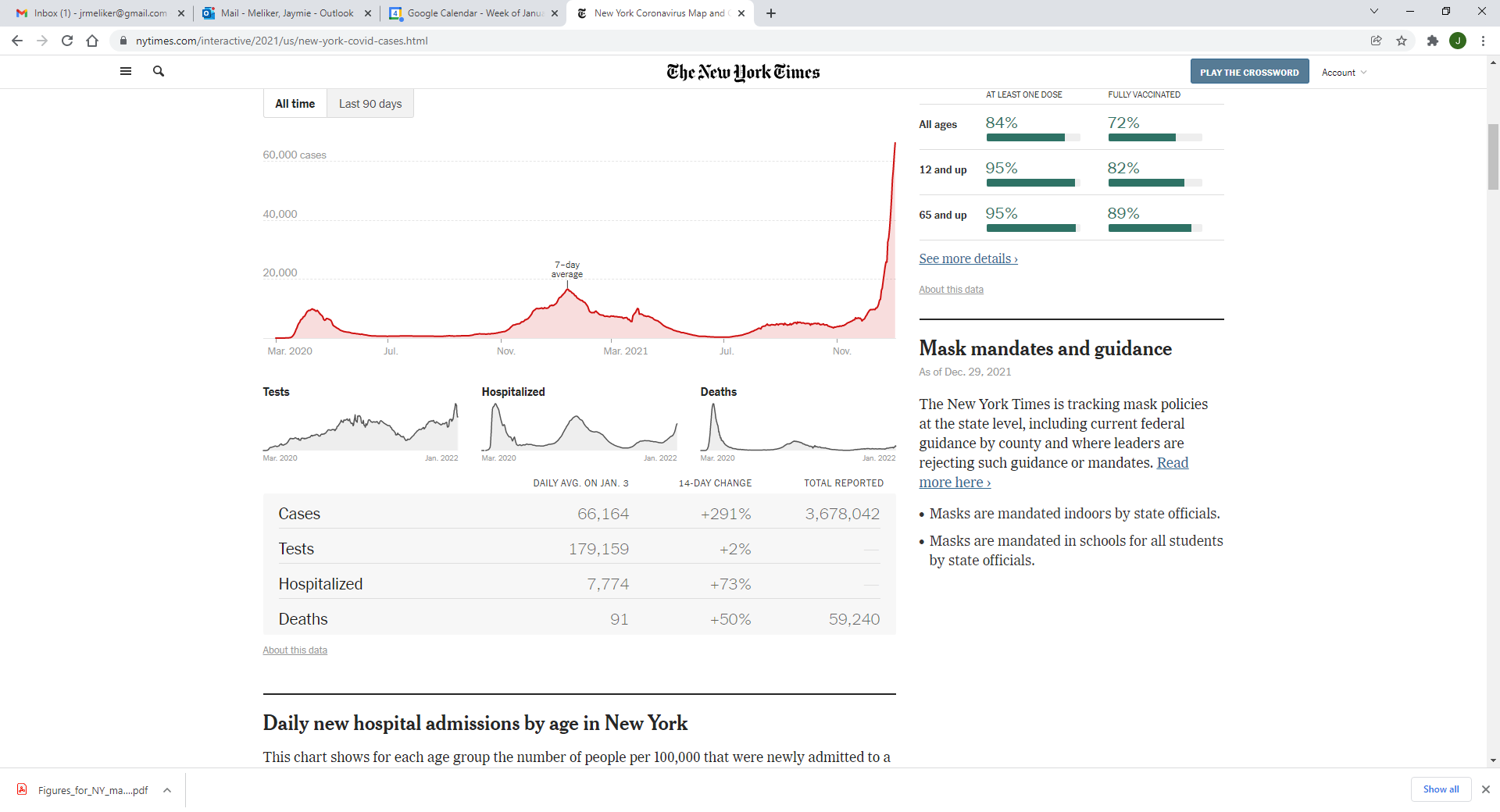


**New York**

**Florida**

**California**


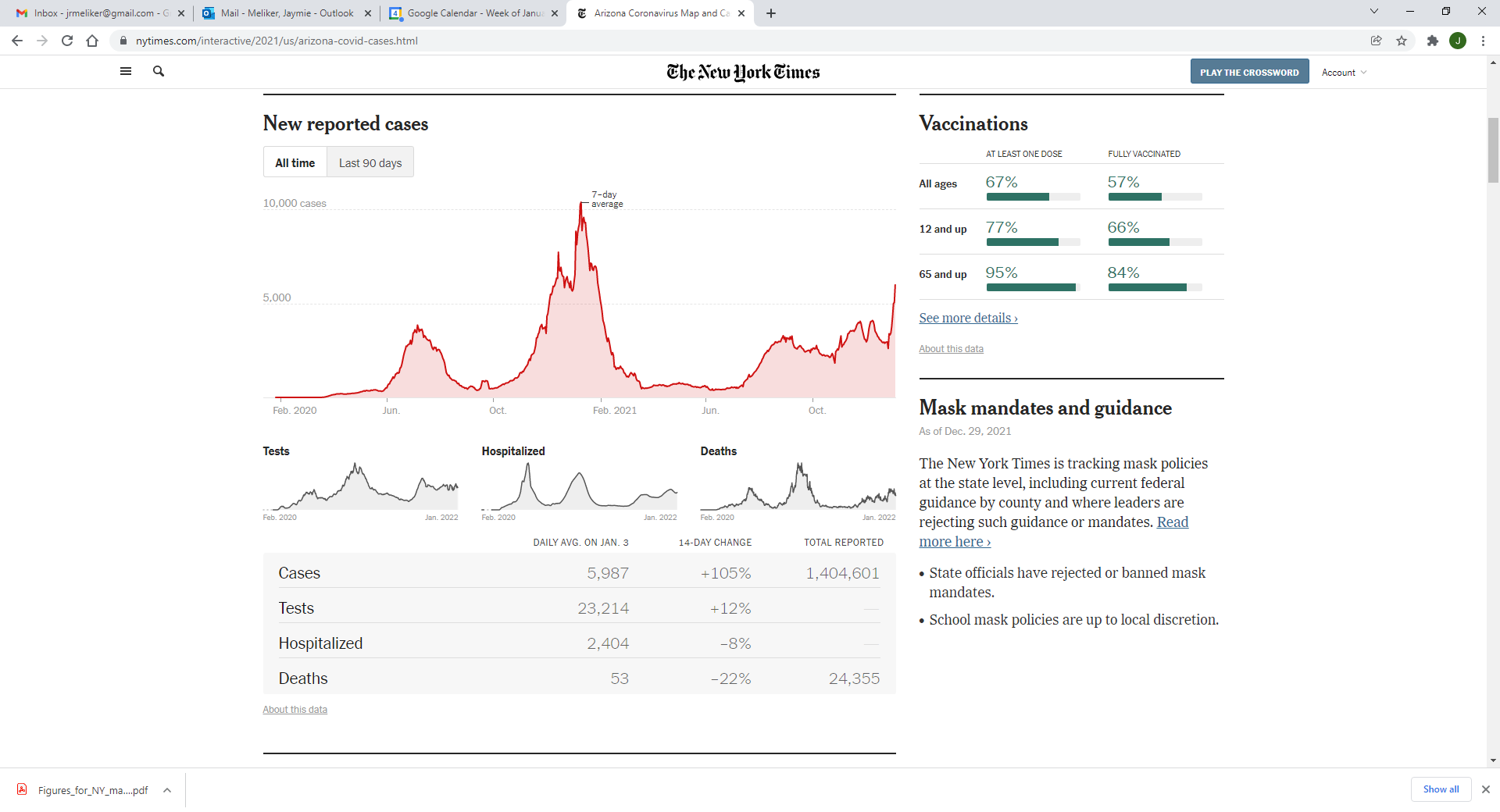

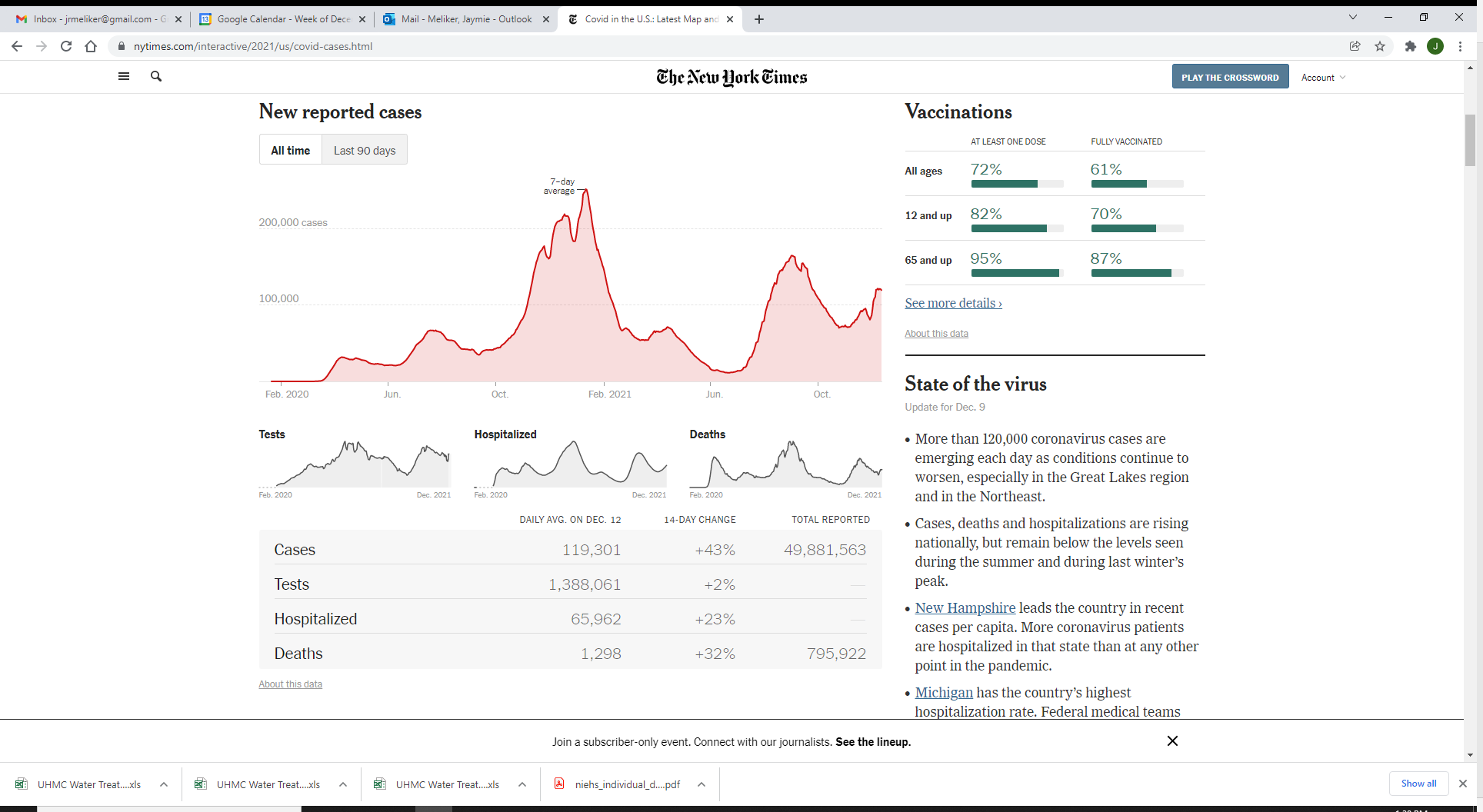


**Arizona**

**United States**

Source: <https://www.nytimes.com/interactive/2021/us/covid-cases.html>. Note the y-axis changes in each figure so that one can visualize the peaks and troughs within each state, but not as clearly across states.

Appendix Figure 1. Google Trends for Change in Depression: Difference between 2020 and the Average of 2018 and 2019, by City, Monthly

Google Trend scores lie between 0 to 100. A change equal to 5 reflects ~5% change in the depression score.

Appendix Figure 2. Google Trends for Change in Anxiety: Difference between 2020 and the Average of 2018 and 2019, by City, Monthly

Google Trend scores lie between 0 to 100. A change equal to 5 reflects ~5% change in the anxiety score.
